# Rare Thyroid Diseases Mimicking or Concealing Primitive Thyroid Neoplasms: A Call for a “Check and Double-Check” Clinical Mindset

**DOI:** 10.7759/cureus.56777

**Published:** 2024-03-23

**Authors:** Frederik Duhamel, Eric Balti, Wim Waelput, Steven Raeymaeckers, Guy Verfaillie, Ine Luyten, Corina E Andreescu

**Affiliations:** 1 Endocrine and Diabetes Unit, Universitair Ziekenhuis Brussel (UZ Brussel), Brussels, BEL; 2 Department of Internal Medicine, Vrije Universiteit Brussel (VUB), Brussels, BEL; 3 Department of Anatomopathology, Universitair Ziekenhuis Brussel (UZ Brussel), Brussels, BEL; 4 Department of Radiology, Universitair Ziekenhuis Brussel (UZ Brussel), Brussels, BEL; 5 Department of Surgical Oncology, Thoracic Surgery and Transplantation, Universitair Ziekenhuis Brussel (UZ Brussel), Brussels, BEL

**Keywords:** primary thyroid lymphoma, anaplastic thyroid cancer, castleman disease, thyroid abscess, thyroid neoplasms

## Abstract

Clinical endocrinologists encounter in their practice patients with thyroid diseases on a daily basis. Still, diagnosis of rare structural thyroid disorders can be quite challenging. In some instances, they do not only impersonate but can also conceal, other conditions such as thyroid carcinomas. We describe a series of patients with structural thyroid disorders including 1) anaplastic thyroid carcinoma initially presenting with features of thyroid abscess; 2) unicentric hyaline vascular Castleman's disease of the thyroid embedded in a stroma of papillary thyroid carcinoma; and 3) primary thyroid lymphoma with a rapid and fulminant evolution. The common challenge in the diagnosis of these cases lies in both their low incidence and their complex presentation. We use the presentation of these cases to raise the attention related to their identification. We highlight the need for precision diagnosis to enable a patient-tailored management approach and improve patient outcomes.

## Introduction

Nodular thyroid pathology encompasses a heterogeneous group including rare thyroid neoplasm. Thyroid ultrasound (US) and US-guided fine-needle aspiration cytology (FNAC) are the most preferred diagnostic tools [1,2]. Thyroid carcinoma is the most common endocrine malignancy, comprising one percent of all cancers, with rising incidence without a change in overall survival rates [3]. While the prognosis of differentiated thyroid carcinoma is generally excellent, the identification of alternative or concurrent differential diagnosis could be qualified to be exotic. We describe and examine a case series of rare structural thyroid disorders concurrently occurring with thyroid neoplasms and a case of primary thyroid lymphoma that could be challenging in daily clinical practice.

## Case presentation

Case 1: thyroid abscess unveiling an anaplastic thyroid carcinoma (ATC)

A 61-year-old Caucasian male with a microprolactinoma treated with cabergoline visited the Endocrinology clinic for painful swelling of the neck persisting for a few weeks, accompanied by hoarseness. He had a tender anterior neck swelling without palpable lymph nodes.

Investigations

The laboratory inflammation markers, except for the C-reactive protein, were within normal range. Thyroid hormone concentration and thyroglobulin were normal (Table 1).

**Table 1:** Overview of reported cases TSH: thyroid-stimulating hormone; TG: thyroglobulin; TI-RADS:  Thyroid Imaging Reporting and Data System; R-CHOP: rituximab, cyclophosphamide, hydroxydaunomycine, oncovin and prednisone. *AJCC/UICC pTNM classification, 8th edition. **Amoxicillin and clavulanic acid.

Characteristics			Case 1	Case 2	Case 3
Age, years			61	21	62
Gender			Male	Female	Male
Ethnicity			Caucasian	North African	North African
Biological parameters at diagnosis					
	TSH (mU/L)	ref. 0.27 - 4.20	0.94	1.39	0.86
	Free T4 (pmol/L)	ref. 11.0 - 24.0	13.2	12.37	14.4
	Free T3 (pmol/L)	ref. 3.1 - 6.8	3.7	4.07	Not available
	TG (mcg/L)	ref. 3.5 - 77.0	22.10	28.2	Not available
	Anti-TG antibodies (kIU/L)	Ref. <115	19	13	17
TIRADS			5	4	5
Bethesda classification			1	3	5
Histology of thyroid resection specimen			Anaplastic thyroid carcinoma with partial squamous differentiation One tumor-free lymph node pT3aN0Mx*	Papillary thyroid carcinoma 4/6 tumor invaded lymph nodes pT3bN1aMx*	ABC type diffuse large B-cell lymphoma With extensive blastic morphology
Molecular pathology			BRAF mutation	BRAF mutation	Not informative
Management			Antibiotics**, right hemithyroidectomy, radiochemotherapy	Total thyroidectomy and radioactive iodine therapy	Two steps total thyroidectomy, chemotherapy and anti-CD20 monoclonal antibody therapy (R-CHOP)
Evolution			Deceased	Complete remission	Deceased

**Figure 1:**
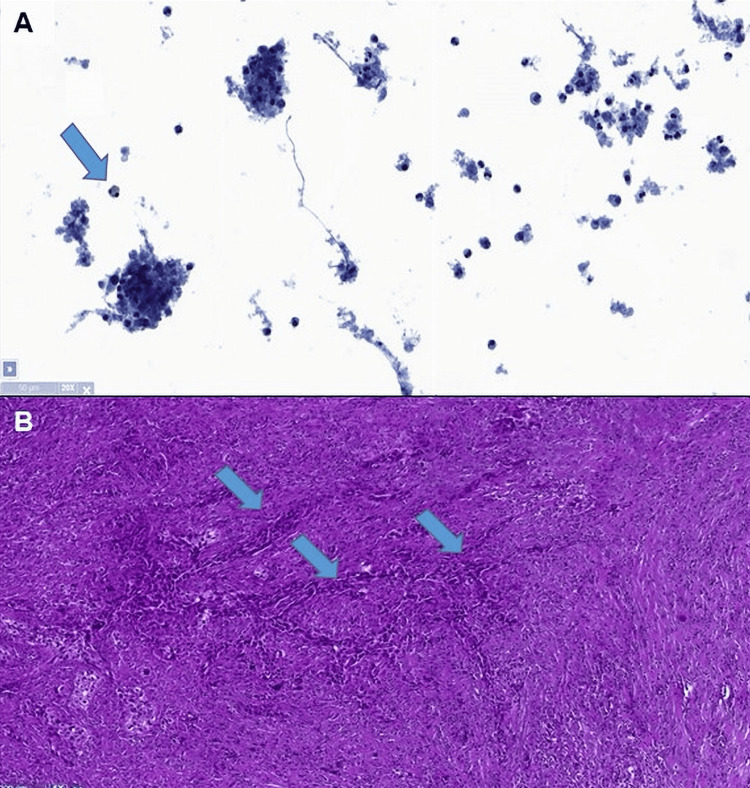
Moderately cell-rich sample with a severely mixed inflammatory infiltrate: numerous neutrophils, few hemosiderin-loaded macrophages (arrow) in a necrotic and fibrinoid background. No presence of epithelial cells. Bethesda classification (2010 Edition): Class I (non-diagnostic) (A); H&E 5x: infiltrative cords of large, atypical cells in the fibrous stroma with a dense mixed inflammatory infiltrate (arrow) (B).

Thyroid ultrasound revealed a plunging goiter with a large hypo-echogenic solid nodule in the right lobe measuring 3.9 cm x 4.0 cm x 4.2 cm with areas of macrocalcifications and a surrounding halo, with an attributed score of TIRADS 5 [1]. US-guided fine-needle aspiration (FNAC) showed a conglomerate of inflammatory cells mainly made of polymorphonuclear cells and a few macrophages in a background of necrotic and fibrinoid tissue suggestive of thyroid abscess. No neoplastic cells were found (Figure 1A). The microbial culture remained sterile.

Treatment

He was admitted and a 10-day antibiotic regimen of amoxicillin/clavulanic acid was administered, and a percutaneous abscess drainage was performed. Reports of cytologic examination and microbial culture of drainage fluid were similar to those of the FNAC. CT examination of the neck revealed a thick-walled necrotizing mass with incipient destruction of thyroid cartilage. A decision was made to proceed with the right diagnostic hemithyroidectomy. During surgery, the necrotic lesions mainly involved the right thyroid lobe. A suppurative collection was evacuated from the lower right lobe. Further exploration revealed an extensive lesion invading the common carotid and the thyroid cartilage.

Histopathology was diagnostic of ATC with partial squamous differentiation invading almost the entire thyroid capsule (6.0 cm) with extrathyroidal extension (Figure 1B) into surgical margins. 18-fluorodeoxyglucose-positron emission tomography (18F-FDG-PET/CT) showed no lymph nodes or distant metastasis. The molecular analysis revealed BRAF V600E activating mutation.

Outcome and Follow-up

Hemithyroidectomy enabled pain control and that of the local swelling. Immunohistochemistry revealed a high proliferation index with 90% positivity for Ki67 and a high expression of p53. Further treatment consisted of concomitant radio-chemotherapy consisting of 30 fractions of two gray combined with a weekly regimen of carboplatin and paclitaxel for five cycles. One month after completing the treatment, the patient presented at the emergency department with difficulty breathing. Neck CT revealed progressive disease with significant compression of the trachea. The patient opted for palliative sedation and passed away shortly after.

Case 2: unicentric Castleman disease (CD) disguised as nodal recurrence of papillary thyroid carcinoma

A 21-year-old woman consulted her primary care physician for a recent sudden swelling of the left lateral side of the neck. She also complained of dysphagia. Clinical examination identified several cervical lymph nodes, which initially were attributed to a recent diagnosis of mononucleosis. Thyroid function tests and thyroglobulin levels were normal (Table 1).

Investigations

Ultrasound of the neck showed a solitary nodule with a diameter of 2.6 cm in the left lobe of the thyroid gland with structural heterogeneity, mild hypervascularity, diffuse microcalcifications, and several surrounding lymph nodes (TIRADS 4). Cytology suggested a follicular lesion of undetermined significance (Bethesda III).

Treatment

**Figure 2:**
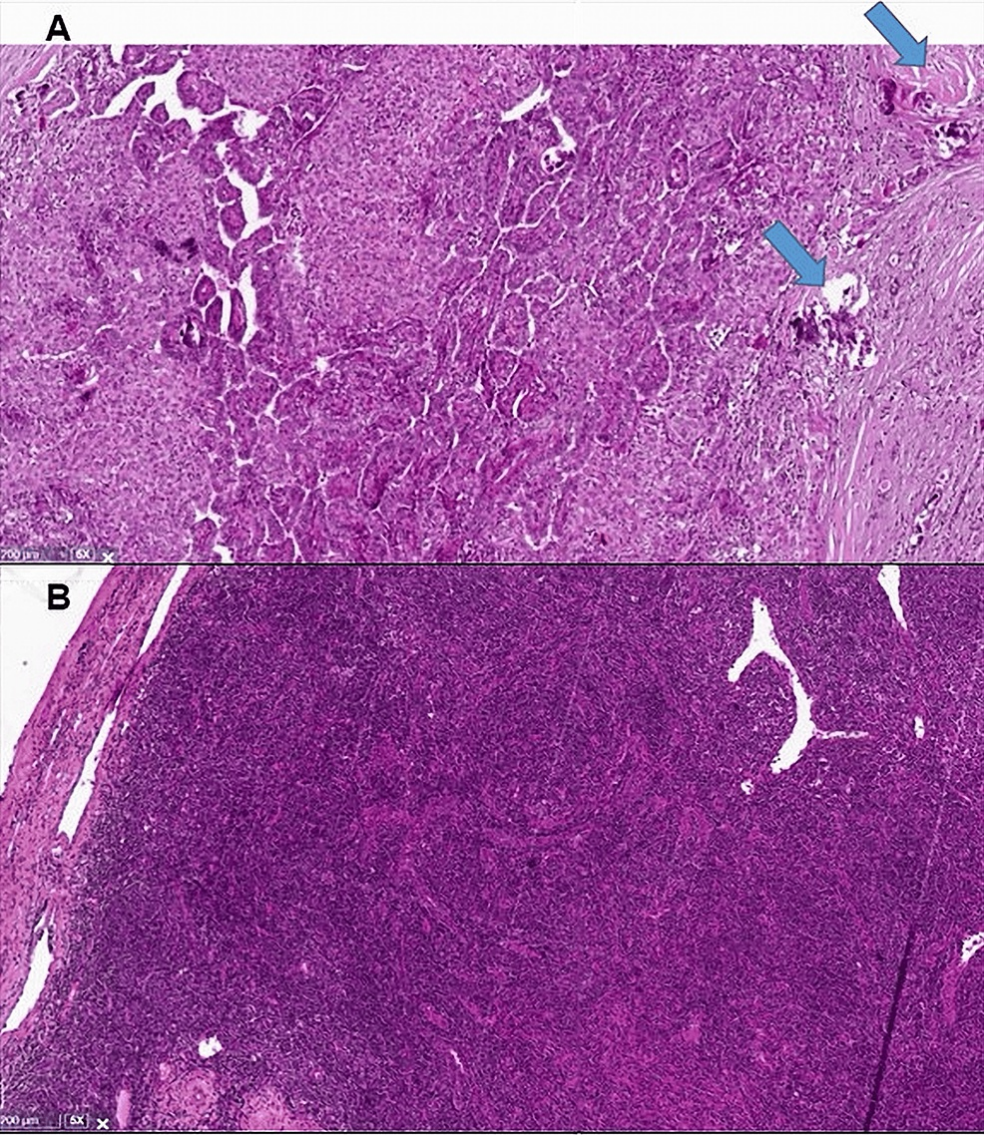
H&E 5x: papillary carcinoma with typical papillary fronds lined by atypical cells with classical nuclear features and several psammoma bodies (arrows) (A); H&E 10x: proliferation of high endothelial venules (B).

A diagnostic hemithyroidectomy was conducted. During surgery, the thyroid nodule appeared firm and depicted macroscopic signs of local inflammation. Intraoperative examination of the left lobe showed a diffuse sclerosing variant of papillary thyroid carcinoma. Routine histology confirmed the finding. Based on the finding, the right thyroid lobe was also removed (Figure 2A) and central cervical dissection was performed. Four of the lymph nodes tested positive for metastasis conferring pT3bN1aMx classification status. Adjuvant radioactive iodine-131 therapy (100 mCi) complemented the treatment.

Outcome and Follow-up

Six months later during a follow-up consultation, a large new lymph node could be felt during palpation of the posterior border of the left sternocleidomastoid muscle (region Vb). Ultrasound confirmed a suspicious node with a diameter of over 5 cm. Additional FNAC was carried out, showing no evidence of metastatic disease.

However, 18F-FDG-PET/CT showed mild metabolic activity in the suspicious lymph node without other abnormalities suggestive of distant metastasis. The lymph node was surgically removed for microscopic examination. Histopathology found altered follicular architecture with prominent vascular proliferation in the interfollicular regions and signs of hyalinization (Figure 2B). The diagnosis of hyaline vascular (HV) Castleman disease (CD) was retained. Human herpesvirus 8 (HHV-8) infection, monoclonal immunoglobulin, T-cell receptor gene rearrangements, and multicentric lymph node involvement were excluded. At the time of this report, the patient was in remission for both papillary thyroid carcinoma and CD.

Case 3: primary thyroid lymphoma

A 62-year-old male was referred to us for a nodule in the right thyroid lobe discovered in the context of mild neck discomfort.

Investigations

**Figure 3:**
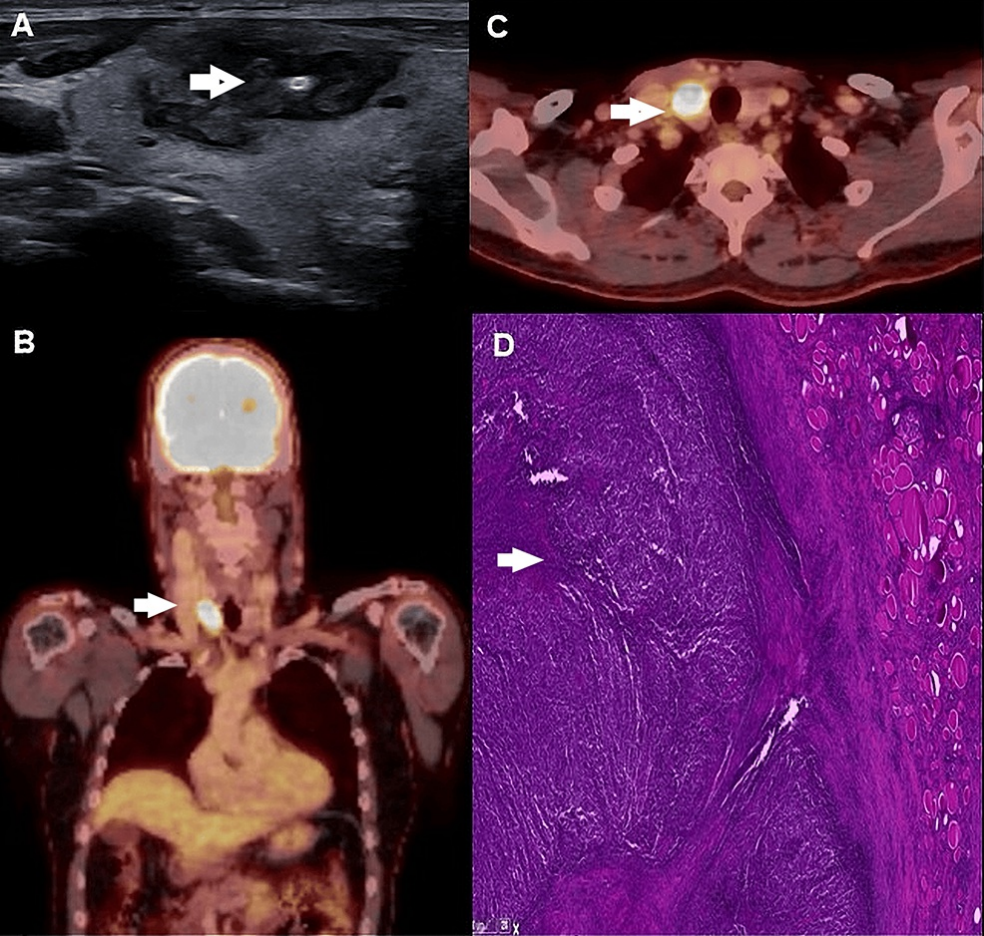
Ultrasonographic image of the right thyroid lobe showing an heterogeneous lesion with a large central hypo echogenic area, irregular margin and a marginal hyper echogenicity (arrow) embedded in the hypo echogenic region. Coronal and axial views of 18-Fluorodeoxyglucose ([18F]FDG) positron emission tomography (PET)/computed tomography (CT) image showing a highly metabolic lesion (arrow) in the right cervical region suggestive of thyroid carcinoma (B, C, respectively);  H&E 2x: Follicular architecture partially effaced by a proliferation of lymphocytes (arrow). These were PAX5/MUM1 positive; the proliferation index (Ki67) was above 90% (D).

Ultrasound revealed a solid, hyperreflective, and irregular TIRADS 5 nodule in the right thyroid lobe. The thyroid function tests were normal. FNAC was performed, suggesting a highly suspicious lesion for malignancy (Bethesda 5) (Table 1, Figure 3A). 18F-FDG-PET/CT showed isolated hypermetabolic activity in the right thyroid with a maximum standard uptake value, suggestive of primary thyroid carcinoma, ruling out distant metastasis (Figures 3B, 3C).

Treatment

A total thyroidectomy was performed. Histopathology revealed an aggressive diffuse large B-cell lymphoma (DLBCL) of the activated B-cell subtype with vascular invasion (Figure 3D). Immunophenotyping showed normal NK cells, T-lymphocytes, and B-lymphocytes with a polyclonal distribution. No abnormalities were found in the myelogram. A lumbar puncture ruled out lymphoma cells in the cerebrospinal fluid. Molecular analysis was negative for monoclonal immunoglobulin gene rearrangements. The lymphoma was staged as IE with a favorable prognostic index score (R-IPI I).

The patient received the R-CHOP (rituximab, cyclophosphamide, doxorubicin, vincristine, and prednisone) systemic chemotherapeutic regimen and achieved a complete metabolic response after four cycles. After multidisciplinary discussion (MDD) two additional cycles of R-CHOP were given, which he tolerated well [4-6].

Outcome and Follow-up

Shortly after completing chemotherapy, the patient presented to the Emergency Department with stridor. His respiratory symptoms were attributed to bilateral pneumonia caused by the SARS-CoV-2 virus. He was admitted to the Intensive Care Unit and was intubated due to acute respiratory distress syndrome. He passed away a few weeks later due to ventilation-associated pneumonia.

## Discussion

We presented two rare thyroid diseases, thyroid abscess and CD of the thyroid, concurrently occurring with thyroid neoplasms and a case of primary thyroid lymphoma. Because of their scarcity, these conditions could be challenging to identify in daily clinical practice. This defines the relevance of their description with the aim to increase awareness of clinicians since the correct and early identification of these conditions impacts the nature and promptitude of the treatment as well as the associated prognosis. Furthermore, an accurate diagnosis has implications for the patient and healthcare costs.

Conspicuous complexity of concurrent rare thyroid diseases

ATC is a rare condition associated with a poor prognosis and high lethality rate. Our patient survived five months after diagnosis which is in line with the median survival of about six months reported in the literature [7]. The diagnostic yield of FNAC is highly variable, but quite often may be nondiagnostic due to sampling issues related to the presence of tumor necrosis and reactive and inflammatory changes as was the case in our patient. Besides the diversity in molecular patterns, the cellular presentation is diverse and can be summarized in three main subgroups including sarcomatoid or spindle cell, giant cell, and squamoid or epithelial [8]. Simultaneous association with differentiated thyroid carcinoma has been described in most anaplastic thyroid cancers harboring BRAF V600E mutation, which was not the case in our patient despite the presence of BRAF V600E activating mutation [8,9]. He initially presented with a thyroid abscess that delayed the diagnosis. The modality of the onset of suppurative thyroiditis remains unclear. However, one could hypothesize that the infection risk could be increased by the high propensity to tumor necrosis described in up to 86% of cases of ATCs [8]. The potential modalities of infection onset are so far speculative. While some support the spread of the infection from a primary site near the thyroid, others hypothesize that an embolic infectious event with hematogenous dissemination could foster the onset of focal infection in the thyroid [10,11]. In our patient, the infection site in the vicinity van the thyroid was ruled out using PET-CT as well as gastroscopy. It is however worth mentioning that this feature is not specific to ATC. It is likely that the absence of an isolated infectious agent is inherent to the high suspicion index of underlying malignancy. There is however lack of sufficient data to support this hypothesis [10]. More data are needed to establish the association of sterile suppurative thyroiditis with oncologic thyroid diseases in general and specifically ATC. Furthermore, coordinating early multidisciplinary involvement of endocrinologists, surgeons, radiation and medical oncologists, and palliative care teams is necessary to arrive at options for best patient care.

The opportunity of serendipitous diagnosis of CD of the thyroid

As previously reported, ATC can conjointly occur with differentiated thyroid carcinoma [9]. Here we report a case of unicentric CD of the thyroid a few months after diagnosis of papillary thyroid carcinoma. CD is a lymphoproliferative condition that could harbor several phenotypic presentations [12] and was first described in 1956 [13]. The most common form of the disease when the thyroid is involved is the unicentric disease. All variants of the CD have been reported in the thyroid with most of the time concurrent onset with a rheumatologic, auto immune, or oncologic conditions such as in our case [14,15]. To the best of our knowledge, there is no reported case of multicentric CD involving the thyroid. The CD must be included in the differential diagnosis of cervical nodal enlargements. The diagnostic problem is because it can be confused with other neoplasms of the head and neck. The primary diagnosis of CD is extremely difficult. We must however acknowledge the relevance of identification of a morphotype of the disease lies in the opportunity to rule out a multicentric disease, potentially associated comorbidities, or predisposing factors and to conduct definitive surgical care in the case of focal disease. 

Primary thyroid lymphoma: a heterogeneous disease

Lymphomas arising from the thyroid are extremely rare. The vast majority are non-Hodgkin lymphomas, which represent 1%-5% of all thyroid neoplasia [4]. The subtype and the disease stage determine the diagnosis specificity and the prognosis. Despite the identification of DLBCL tends to be relatively easy on cytology, the diagnosis sensitivity of thyroid lymphoma in general, after fine needle aspiration, is increased by the combination of cytology, cell block analysis, and immunohistochemistry [16]. The tendency for the disease to occur more frequently in patients with long-standing autoimmune diseases including, those of the thyroid, particularly is remarkable [17]. This is well illustrated by the genetic signature of the more indolent extra-nodal marginal zone lymphoma of thyroid mucosa-associated lymphoid tissue that has been shown to harbor deleterious mutations affecting tet methylcytosine dioxygenase 2 (*TET2*), cluster of differentiation 274 (*CD274* aka *PD-L1* gene) and tumor necrosis factor receptor superfamily member 14 (*TNFRSF14*) genes in most of the cases [18]. However, the presence of* CD274* (*PD-L1*) gene mutation seems not to impact PD-L1 expression on immunohistochemistry. Whether or not these mutations have an impact on the behavior of immune cells and treatment response warrants however further investigation.

## Conclusions

We describe three cases that could be challenging in daily clinical practice because of their shared presentations with other thyroid diseases. Accurate histopathological examination takes a canonical place in their diagnostic adjudication, guiding appropriate treatment decisions and averting unnecessary interventions. A multidisciplinary approach is essential to ensure accurate diagnosis and tailored treatment. Further research is however needed for a better understanding of these rare thyroid conditions, to improve patients' care, and by so doing their outcomes.
